# Early Postoperative Weight Bearing Following the Reconstruction of Traumatic Complex Tibial Bone Defects by Vascularized Free Fibula and Ilizarov External Fixator: A Case Series

**DOI:** 10.7759/cureus.74207

**Published:** 2024-11-22

**Authors:** AbdAllah Elgouhary, Mohammed A Sanad, Ahmed I Elgammal, Ahmed Elsayed, Chaitanya Dodakundi, Khalid A Alawadi, Hamed A Badawi

**Affiliations:** 1 Department of Orthopedics and Traumatology, Rashid Hospital, Dubai, ARE; 2 Department of Orthopedics and Traumatology, Sheikh Khalifa Medical City, Abu Dhabi, ARE; 3 Department of Hand and Microsurgery, Rashid Hospital, Dubai, ARE

**Keywords:** bone defect, fibula, illizarov, pedicled, vascularized

## Abstract

Objective: Post-traumatic tibial bone defects represent a significant challenge to orthopedic surgeons. Various reconstructive methods are available based on associated local soft tissue injury and defect size. Free vascularized fibular graft represents a major successful technique; combined with a rigid Ilizarov external fixator, it allows safe, immediate postoperative weight bearing. In this article, we describe a series of six patients managed according to the previously presented plan, achieving satisfactory results.

Methods: A series of six patients, all males with an average age of 33.3 years, underwent reconstruction for post-traumatic complex tibial bony defects using contralateral free vascularized fibular grafts and Ilizarov external fixation. Initially, all patients underwent multiple sessions of debridement and a simple pin-to-bar external fixator. The bony defect averaged 15 cm, and the average harvested length of the fibula used for reconstruction was 22.1 cm. All patients started immediate total weight bearing postoperatively, with a mean time of 17 days after bony union Ilizarov was replaced with minimally invasive plate osteosynthesis (MIPO) in all patients, and continued full weight bearing (FWB).

Results: During the follow-up period, averaging 19.3 months, all patients achieved bony union with a mean time of 3.75 months. Patients spent an average of 6.4 months in the Ilizarov frame before it was replaced with MIPO; graft hypertrophy occurred in all patients, averaging 52.6%.

Conclusion: The combined use of a vascularized fibular bone graft and an Ilizarov frame proves to be a successful and safe approach for immediate postoperative FWB. This yields comparable outcomes in terms of union and function.

## Introduction

Treatment of traumatic skeletal and soft tissue defects represents a significant challenge to an orthopedic surgeon, especially leg trauma, considering the subcutaneous nature of the tibia, minimal soft tissue coverage, and liable vascular supply with poor healing and non-union potentials. Various reconstructive methods are available, including conventional bone grafts. Each has advantages and disadvantages. However, when the bone defect is larger than 6 cm, has a severe soft tissue defect, or is infected non-union, more specialized techniques such as bone transport and vascularized fibular graft (pedicled or free) become more essential [1-5].

Bone transport, or distraction osteogenesis, using an external fixator is an option to treat large bone defects. Preoperative patient education is necessary to increase compliance with treatment, as the treatment is lengthy and painful and associated with complications. Although some surgeons had excellent results with this technique, others found high failure rates, a higher number of secondary procedures for non-union at the docking site, regeneration, and prolonged healing time [6-8].

Fibula is the most common vascularized bone graft used; it can provide bone length up to 26 cm without significant donor site morbidity [1]. It retains its intrinsic blood supply and bypasses the creeping substitution process, which characterizes the healing of avascular grafts and converts bony defects to segmental fractures. As a result, it hastens bone healing and causes hypertrophy [9]. This, coupled with dense cortical bone and triangular cross-sectional area, provides mechanical support that resists axial and rotational stresses [10]. In traumatic defects, an ipsilateral fibula is usually found to be missed or fractured, or if soft tissue reconstruction is needed, a contralateral free fibula may be used.

Combining this with the Ilizarov external ring fixator provides a stable yet dynamic system that allows both axial micromotion and compressive loading at the graft site that can begin immediately after surgery. This micro-motion and axial loading favor bone healing and coincide with the current standard of care in orthopedic early mobilization [1,10,11].

Mostly, all literature agreed about the long time needed for union, with an average of three to nine months, starting with partial weight bearing and finally full weight bearing (FWB) after the radiological finding of graft hypertrophy and union. Semaya et al. reported that the mechanical advantages of the Ilizarov frame allow patients to start weight bearing as early as possible, but that was only after signs of the radiological union [1].

In the prospective study described in the present paper, we investigate the result of the combined plan of very early postoperative FWB following the reconstruction of traumatic complex tibial bone defect by free osteocutaneous fibula flap and Ilizarov. In this case series, we describe six male adult cases managed according to the previously presented plan, achieving satisfactory results.

## Materials and methods

This study was approved by the Institutional Review Board (IRB) under the post-graduate medical education section of the Mohammed Bin Rashid University of Medicine and Health Sciences, United Arab Emirates.

This prospective study included six consecutive patients (six tibiae) who underwent reconstructive procedures of large tibial defects as post-traumatic or infected osteosynthesis using a vascularized contralateral free fibular graft with an Ilizarov external fixator between 2018 and 2023. All patients were males with an average age of 33.3 years (18-42 years). The initial injury in four patients was an open fracture. Three patients were Gustilo grade IIIb, involving the middle third of the tibia with or without fibula fracture, and one case was grade IIIC involving the middle third as well. The remaining two patients are postoperative infected osteosynthesis of proximal and distal tibiae. The distal tibia was an immediate postoperative surgical site infection with frank pus, which was initially an open fracture GII. However, the proximal tibia was infected with nonunion with double plating after 18 months of the surgery.

Surgical technique: debridement/removal of implant/conventional external fixator

All patients underwent multiple sessions of extensive debridement and removal of all necrotic non-viable bone and soft tissues, removal of implants from infected cases when infection was persistent; one patient with grade IIIC had a vascular injury to anterior tibial artery, which was irreparable and ligated on the initial presentation by a vascular surgeon. Stabilization was achieved with a simple half-pin, pin-to-bar external fixator construct with negative-pressure dressing as temporary measures added when needed. An antibiotic-impregnated polyhydroxyethyl methacrylate (PMMA) was used as a temporary spacer in the proximal tibia-infected case. After all sessions of debridement, all tibial defects were large with an average defect of 15 cm (range: 11-19 cm) complicated with skin and soft tissue defects average of 216 cm² (range: 50-375 cm²) (Table 1). On average, it took 12 days from the initial injury to undergo the index procedure, except for the cases that got infected, which took 18 months (proximal tibia) and 47 days (distal tibia) to undergo the procedure after the complete resolution of the infection. The resolution of the infection was determined based on the clinical picture, inflammatory markers, and culture results. Before the index procedure, all patients had CT angiography done for both lower limbs routinely to delineate anatomy, exclude vascular injury, and plan anastomosis site. Preoperative, intra-operative, and postoperative data was collected for comparison.

**Table 1:** Preoperative data

No.	Sex/age (years)	Site	Ipsilateral fibula	Defect length (cm)	Type of fracture	Skin defect (cm)	Time from injury to index procedure	Preceding procedures
1	M/18	Middle third (left)	Loss	12 cm	IIIC	25*15	7 days	Debridement and uniplanar external fixator
2	M/42	Proximal third (left)	Intact	15 cm	Infected	none	18 months	Open reduction internal fixation/implant removal/multiple debridements and cement spacer
3	M/36	Middle third (right)	Fractured	15 cm	IIIB	12*20	6 days	Debridements and uniplanar external fixator
4	M/39	Middle third (right)	Fractured	18 cm	IIIB	15*28	25 days	Debridement/uniplanar external fixator/Ilizarov
5	M/37	Middle third (left)	Intact	19 cm	IIIB	10*20	10 days	Debridement/uniplanar external fixator
6	M/28	Distal third (right)	Fractured	11 cm	Infected open fracture GII	10*5	47 days	Open reduction internal fixation/implant removal/multiple debridements and external fixator

Surgical technique: fibula osteoseptocutaneous free flap and application of Ilizarov

All cases are done under general anesthesia, considering it is a lengthy operation with an average of 9.8 hours (range: 7.5-12 hours). The contralateral fibula was harvested because the ipsilateral fibula was either fractured or associated with soft tissue injury. A tourniquet was used, and the fibula was harvested as an osteoseptocutaneous free flap through a standard posterolateral linear approach. The skin paddle was attached to the posterior intermuscular septum, which contains septal perforators originating from peroneal vessels to supply the skin paddle. The skin paddle had the advantage of providing tension-free soft tissue coverage for skin and soft tissue defects and as an indicator of flap viability in the postoperative period. The whole fibula length was always removed to facilitate peroneal pedicle dissection, leaving at least 6 cm distally to preserve the syndesmotic complex and ankle stability and 3 to 4 cm proximally to maintain lateral knee stability. The graft was then trimmed to the desired length, considering the bone length inlay within the tibial canal. Simultaneously, another team was preparing the receipt site, and the external fixator was adjusted when obstructing the operative field. Both ends of the tibia were refreshed till bleeding edges. Both ends of the fibula were fixed to the recipient's bones by intramedullary doweling at least 2 cm with transfixing screws of 3.5 mm, two screws on each side. Then, anastomosis was made either end to end or end to side through the same or different approach based on the local injury pattern. In two cases a flow-through anastomosis was made to overcome the local soft tissue injury pattern. This anastomosis is made by connecting the free end of the pedicular artery to the proximal stump of the feeding artery end to end, and the distal stump is connected end to side to the pedicular artery. This type of anastomosis was made only in case of conventional anastomosis was not possible. The average length of bony defect is 15 cm (range: 11-19 cm), and the average harvested length of the fibula was 22.1 cm average (17-25 cm) (Table 2). Occasionally, an additional skin graft was needed to cover the remaining defects from the ipsilateral or contralateral thigh.

**Table 2:** Intra-operative data

No.	Harvested length (cm)	Size of skin pedal (cm)	Operative time (hr)
1	20	10*8	12
2	23	10*12	7.5
3	24	12*12	10.5
4	25	12*8	9
5	24	11*20	8
6	17	15*8	12

Then, the conventional external fixator was removed, and the Ilizarov frame was applied as a definitive fixation method to allow immediate postoperative FWB. The frame consisted of two full Ilizarov rings, one attached to the proximal segment of the tibia and fixed to the bone by one reference. Ilizarov wire is 1.8 tensioned to 130, and two half pins are 5 mm, one anterolateral and one anteromedial, putting into consideration the maximum spanning of pins to hold the longest portion of bone and protecting the anastomoses of the fibular graft. The distal ring was fixed to the bone by two Ilizarov wires, one reference and the other transfixing the distal fibula to the tibia. To increase the ring stability, one anteromedial half pin of 5 mm was also added to increase the length of the fixed segment. Both rings are connected with four Ilizarov rods with equal spacing, giving 360 controls on the bone segments. They were allowing adjacent joints to mobilize freely.

Postoperative protocol

Patients were shifted to the surgical ward high dependency unit. In the early postoperative phase, strict limb elevation and bed rest were initiated; flap perfusion was evaluated every two hours through a window made in the dressing by monitoring the skin flap paddle for color, temperature, turgor, capillary refill, and Doppler signal for the anastomosis site. Our surgical strategy allowed all cases to start total weight bearing 15 days post-surgery with an assistive device. This (15-day) time interval is only to avoid the stress of venous congestion that might happen during weight bearing and endanger flap viability. On long-term follow-up, patients were followed for bony union. Moreover, it was assessed as uninterrupted external bony borders between the fibular graft and the recipient's bone in at least three cortices. Once the union was achieved, the Ilizarov fixator was removed in all cases and replaced by long plate fixation LCP 4.5 using the minimally invasive plate osteosynthesis (MIPO) technique, and patients continued weight bearing. The amount of final hypertrophy was calculated based on the formula described by El-Gammal et al.\[ \text{Hypertrophy %} = \frac{\left(\frac{F_2}{R_2} - \frac{F_1}{R_1}\right) \times 100}{\frac{F_1}{R_1}} \]where F_1_ represents the mean fibular graft anteroposterior and lateral width at the midpoint postoperatively, R_1_ represents the mean recipient bone anteroposterior and lateral width at a fixed point away from the graft-host junction postoperatively, F_2_ represents mean fibular graft anteroposterior and lateral width at the midpoint at follow-up, and R_2_ represents mean recipient bone anteroposterior and lateral width at a fixed point away from the graft-host junction at follow-up.

## Results

All our patients were followed regularly; the mean follow-up period was 19.3 months (10-27 months). Primary bony union was achieved after our treatment in all patients (100%) at the proximal and distal junctions. The mean time to union was 3.75 months (3-5 months) (Table 3).

**Table 3:** Postperative data

No.	Time to full weight bearing (d)	Time to removal of Ilizarov (m)	Time to union (m)	Final hypertrophy %	Follow-up duration (m)	Complications
1	13	6	3	62	10	Big toe in plantar flexion
2	11	6	5	47	17	None
3	12	11	4	55.5	21	Underwent revision of congested flap (vein thrombosis)/excision of skin pedal and hemisoleus rotational flap with skin grafting
4	23	5.5	3.5	56	20	None
5	26	5	4	50	27	Stress fracture
6	54	5	3	45	21	None

All patients started immediate FWB with assistive devices after the initial period needed for the anastomosis healing. The mean time to FWB was 17 days (range: 11-26 days); one patient only started FWB after 54 days because of other associated injuries, including chest trauma that delayed rehabilitation. All patients had their Ilizarov ring fixator removed after evidence of bony union, with a mean value of 6.4 months (range: 5-11 months), and replaced by long plate fixation utilizing the MIPO technique. The patient continued FWB.

Graft hypertrophy occurred in all patients. Final graft hypertrophy at the end of follow-up was calculated based on the formula described by El-Gammal et al., with graft hypertrophy with a mean of 52.6% (range: 45%-62%).

Local complications occurred as local pin-tract infection was treated with a regular dressing and oral cefuroxime; no deep infection recurred. One patient had a stress fracture while on the Ilizarov external fixator; there has been no donor site morbidity in any patient. One patient had plantar flexion of the great toe. Another patient developed venous thrombosis of the free flap on postoperative day 7, manifested clinically via flap congestion with preservation of arterial Doppler signal. It was revised, the skin paddle was excised, and a hemisoleus rotational flap with skin grafting was done. A 100% flap survival rate and a bony union were achieved.

At the latest follow-up, no patient reported pain in the reconstructed extremity. All patients had a full range of motion of the adjacent joints and could return to their daily activities, including work.

## Discussion

Managing large bone defects of tibiae represents a major and complex clinical and surgical challenge to the orthopedic surgeon, especially after trauma when compounded with soft tissue loss and vascular injury; this necessitates a well-coordinated approach between orthopedic and reconstructive surgeons to achieve the best possible outcome [1-5].

Different reconstructive options have been described. When a defect is less than 6 cm, a non-vascularized autogenous bone graft is the preferable method; however, even with this minor defect, associated traumatic and soft tissue injuries may devitalize surrounding structures and make the bed of the defect and neighboring soft tissues suffer poor vascularity and scarring and contribute to graft failure [9,12]. More specialized techniques are necessary in these situations or when defects are larger than 6 cm.

The free fibular graft was quickly adopted to reconstruct critically sized traumatic bone defects, especially in these complex situations, through the simultaneous import of bone and soft tissue coverage. The significant advantage of this technique over others is that it can provide up to 26 cm of bone graft that preserves its dual blood supply derived from periosteal and endosteal blood vessels, which transforms the defect into a segmental fracture [13,14], resulting in a graft that bypasses the process of creeping substitution, which is characteristic of avascular grafts, and enhances union and hypertrophy along with its more excellent resistance to infection [15]. This by default maintains the biomechanical architecture and, when combined with rigid internal/external fixation, allows safe weight bearing. Allowing patients' early mobilization and weight bearing is a critical component of trauma management.

Considering the size and shape mismatch between the fibular graft and recipient site, especially at the metaphyseal junctions, choosing the type of fixation is critical to achieving this goal. Semaya et al. reported the advantage of combining free fibular graft with Ilizarov external fixation to allow immediate weight bearing; however, they achieved FWB after an average of 7.3 months from the index procedure after the bony union.

Ilizarov frame allows compression at the segment graft interface, which enhances bone healing and prevents the other three forces (tension, torsion, and bending), which protect the long fibular graft from breakage due to these forces [16]. This advantage allows early weight bearing, which preserves the proprioception of the patient's ankle and knee range of movement and prevents osteoporosis due to prolonged non-weight bearing. All patients at the end of treatment have no muscle atrophy or disuse wasting.

**Figure 1:**
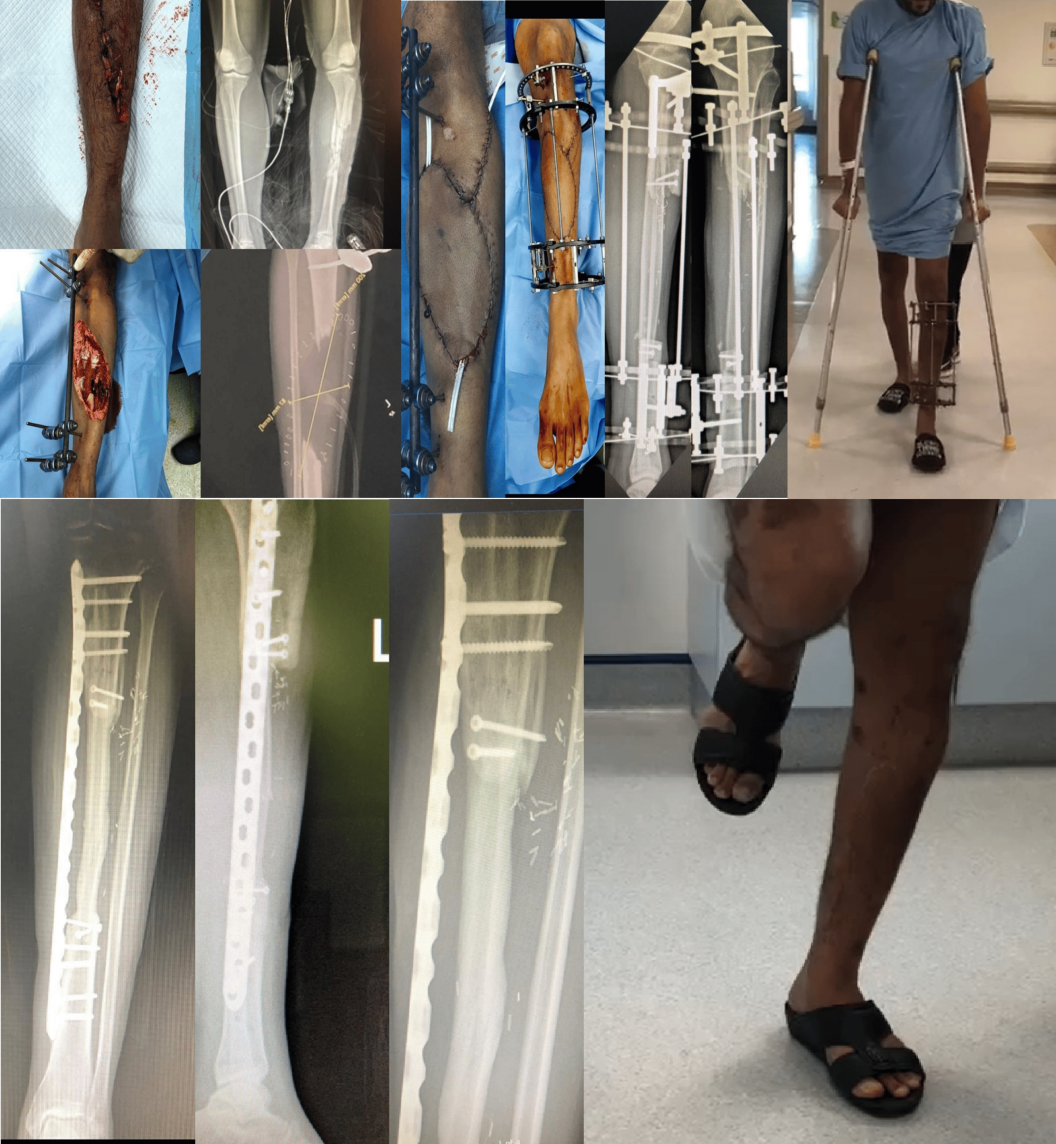
Case #4-grade IIIB right tibia fracture defect size 18 cm. Ten days after the injury, the patient underwent a free fibula graft 24 cm long and applied an Ilizarov external fixator. The patient started weight bearing 26 days after the surgery. Five months after the index procedure, the Ilizarov was removed and replaced with the 4.5 mm minimally invasive plate osteosynthesis technique, and continued full weight bearing

**Figure 2:**
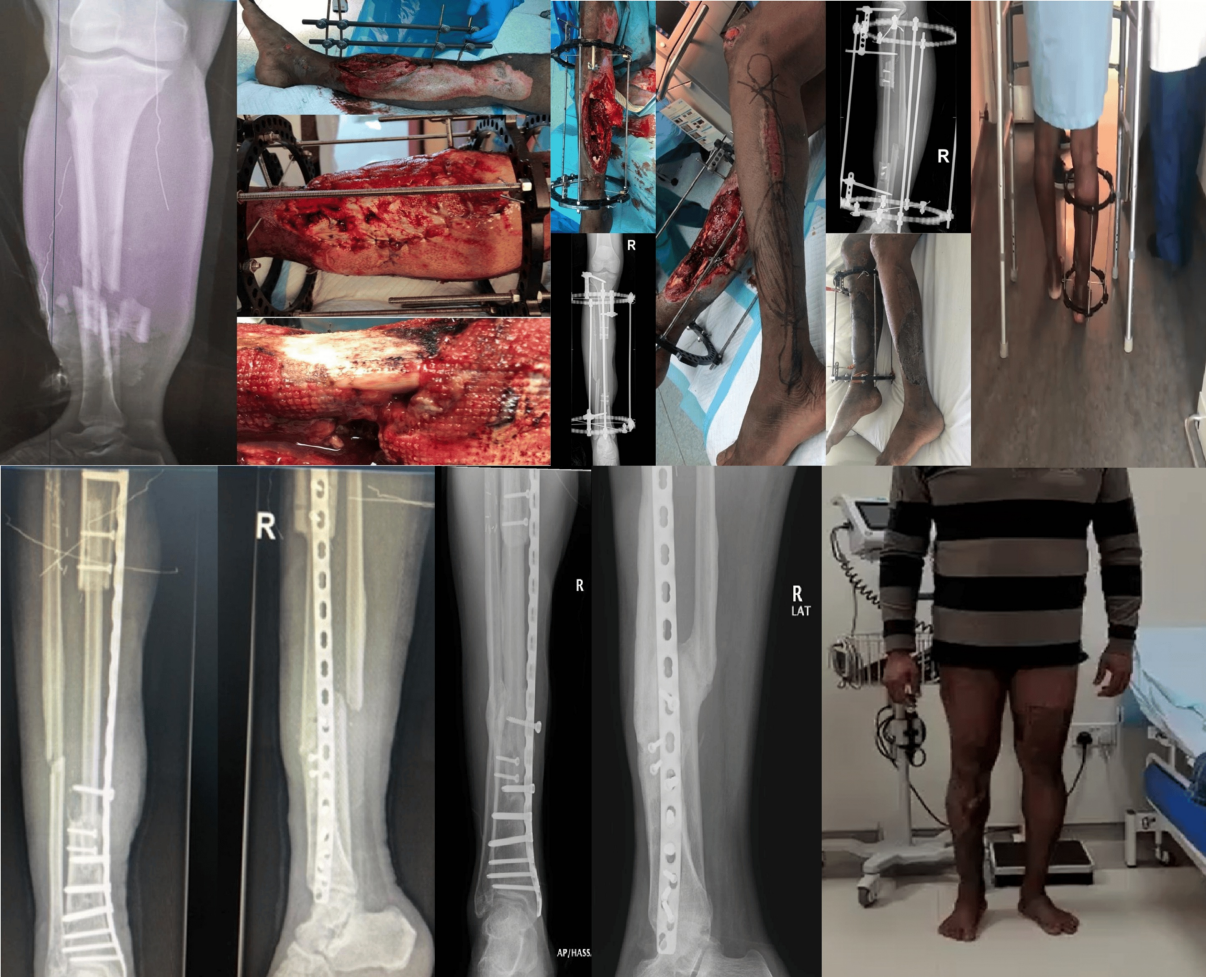
Case #5-grade IIIB left tibia fracture defect size 19 cm. Twenty-five days after the injury, he underwent a free fibula graft, 25 cm in length, along with the application of an Ilizarov external fixator. The patient started weight bearing 23 days after the surgery. Five and a half months after the index procedure, Ilizarov was removed and replaced with the 4.5 mm minimally invasive plate osteosynthesis technique, and full weight bearing continued

The primary purpose of this article is to present our thoughts that combining a free fibular graft with a rigid Ilizarov external fixator allowed immediate postoperative weight bearing. This allows micromotion that promotes bony healing and exposes the fibula to continuous mechanical stresses, which is thought to be the main factor in promoting hypertrophy [17-19]. We chose to combine a fibular graft with Ilizarov external fixation with a configuration to allow this goal; full unprotected weight bearing was commenced on mean time 17 days (range: 11-26 days) in five cases only after the vascular anastomosis healed to avoid the stress of venous congestion that might happen during weight bearing and endanger flap viability. We achieved a 100% union rate on a mean time of 3.75 months (range: 3-5 months) compared to 3.9 months in other reports [1], and final graft hypertrophy of mean 52.6% (range: 45%-62%) calculated based on the formula described by El-Gammal et al. (Figures 1-2) [20]. This could be related to the continuous mechanical load performed by early weight bearing and rigid external fixation configuration that allows micromotion that stimulates biological healing. The Ilizarov external fixator was removed on a mean time interval of 6.4 months after bony union compared to an average of 5.9 months [1] and replaced by long plate fixation using the MIPO technique on the same setting of Ilizarov removal, and the patient was allowed to continue FWB, avoiding keeping the patient without any fixation at any specific time; this might explain the low incidence of stress fractures reported in our series, 16.7%, which happens only when repetitive mechanical stresses exceed the bone strength [10], compared to 25% in other reports [10,18].

## Conclusions

In conclusion, all our patients had a limb salvage rate of 100%, and all returned to preinjury ambulation status and work. Free fibula graft combined with Ilizarov external fixation with stable configuration allows immediate unprotected weight-bearing mobilization in the postoperative period even before evidence of radiographic signs of bony union or graft hypertrophy.
